# Temporomandibular Joint Involvement Revealing Psoriatic Arthritis: A Case Report

**DOI:** 10.7759/cureus.69580

**Published:** 2024-09-17

**Authors:** Imane Bensaghir, Latifa Tahiri, Sara Farih, Hanan Rkain, Fadoua Allali

**Affiliations:** 1 Department of Rheumatology B, Ayachi Hospital, Ibn Sina Hospital Center, Faculty of Medicine and Pharmacy, Mohammed V University, Rabat, MAR; 2 Department of Exercise Physiology and Autonomous Nervous System, Faculty of Medicine and Pharmacy, Mohammed V University, Rabat, MAR

**Keywords:** adalimumab (humira), case report, methotrexate, psoriatic-arthritis, temporomandibular joint-tmj, tumor necrosis factor-alpha (tnf-α) inhibitors

## Abstract

Temporomandibular joint (TMJ) involvement in psoriatic arthritis (PsA) is infrequent but can lead to significant pain and challenges in effective mouth opening or closing. In this report, we present a clinical case of a patient with TMJ involvement revealing PsA.

The patient is a 35-year-old male with a history of guttate psoriasis, who presented with several weeks of TMJ pain and decreased mouth opening, followed by inflammatory lumbar, buttock pain and oligoarthritis. The diagnosis of PsA with TMJ involvement was established based on clinical manifestations per the ClASsification criteria for Psoriatic ARthritis (CASPAR) criteria. Full remission was achieved with tumor necrosis factor alpha inhibitors.

Frequently overlooked or untreated, delayed diagnosis contributes to extensive deterioration of TMJ structures, resulting in persistent discomfort and a detrimental impact on the patient's quality of life. Therefore, prompt initiation of appropriate treatment is crucial to enhance patient well-being.

## Introduction

Psoriatic arthritis (PsA) is a type of seronegative, chronic, inflammatory arthritis that is associated with psoriasis [1]. As the disease progresses and affects multiple joints, patients may exhibit a variety of clinical symptoms. The most common parts of PsA afflicted are the fingers, spine, and nails. People of both genders are equally affected, and those between the ages of 40 and 50 make up the majority of cases [2]. Because the condition can resemble reactive arthritis, rheumatoid arthritis, and ankylosing spondylitis, a thorough examination is essential for accurate diagnosis and proper treatment [1].

In some rare cases, PsA can affect the temporomandibular joint (TMJ), which can cause a lot of pain and difficulty opening or closing the mouth effectively. TMJ involvement in PsA usually goes undiagnosed or untreated resulting in irreversible damage to the temporomandibular joints.

In this report, we present the case of a Moroccan patient with TMJ involvement revealing psoriatic arthritis.

## Case presentation

The patient is a 35-year-old male who was diagnosed with guttate psoriasis six months ago received topical treatment and achieved remission. He also had a family history of a father with seronegative rheumatoid arthritis. 

The patient presented with several weeks of bilateral TMJ pain and decreased mouth opening, followed by inflammatory buttock pain, especially on the left side and inflammatory lumbar pain. Physical examination revealed an oligoarthritis involving the first metacarpophalangeal joint on the left, the right knee and the fifth metacarpophalangeal joint on the right.

Blood tests revealed an accelerated erythrocyte sedimentation rate (ESR) of 50 mm at the first hour and a C-reactive protein (CRP) of 43.9 mg/l. Anti-cyclic citrulline peptide (anti-CCP) antibodies and rheumatoid factor (RF) were negative (Table 1).

**Table 1:** Blood test screening for inflammatory markers and autoimmune conditions

Test	Result	Reference range	Measuring unit
Erythrocyte sedimentation rate (ESR)	55	≤15	mm/hr
C-reactive protein (CRP)	43.9	≤6	mg/l
Anti-cyclic citrulline peptide (Anti-CCP)	0	< 20	U/ml
Rheumatoid factor (RF)	0	<20	U/ml

**Figure 1:**
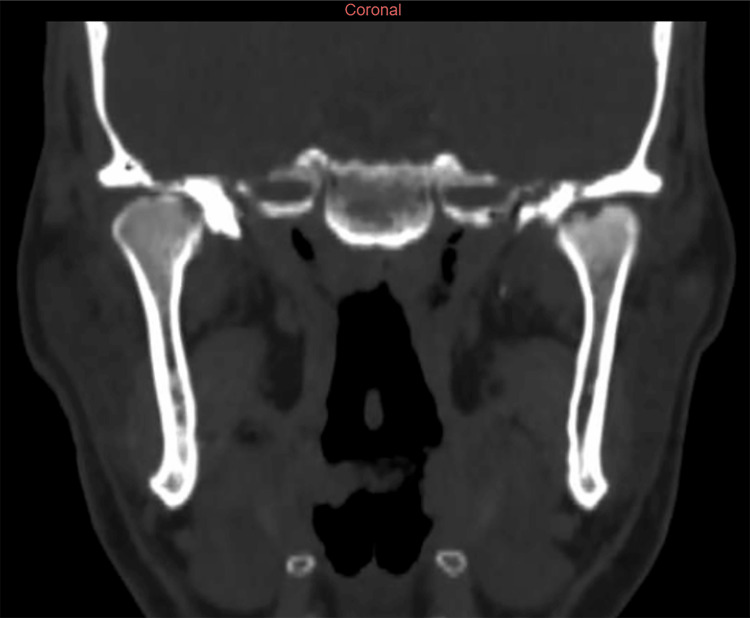
CT scan image of the patient’s TMJ showing bilateral condylar and temporal cortical erosion with joint space narrowing TMJ: Temporomandibular joint

Magnetic resonance imaging of the pelvis showed a right gluteal enthesopathy, and a TMJ CT scan showed bilateral condylar and temporal cortical erosion, suggesting an inflammatory origin (Figure 1).

The diagnosis of psoriatic arthritis with TMJ involvement was established based on clinical manifestations per the CASPAR criteria and radiographic features.

The patient was followed concomitantly in rheumatology and in maxillofacial surgery and was prescribed non-steroidal anti-inflammatory drugs (NSAIDs): etoricoxib 90mg daily and methotrexate 25mg weekly. 

At 15 weeks of taking methotrexate, we observed an improvement of the arthritis and of the inflammatory markers (ESR and CRP) but the TMJ pain was dependent on the daily intake of etoricoxib: each time the patient stopped taking the NSAID, he felt a relapse of the TMJ pain. Therefore, the TMJ involvement was considered refractory to methotrexate and treatment with a TNF inhibitor was indicated: adalimumab 40mg injections every two weeks.

At the six-month evaluation, the patient had achieved full remission under adalimumab 40mg every two weeks and methotrexate 25mg weekly. A follow-up CT scan showed a stabilization of the TMJ lesions and no further structural damage.

## Discussion

Clinical characteristics of psoriatic arthritis affecting the TMJ include disrupted occlusion, discomfort, swelling near the ear, jaw deviation during opening and closing, limited mouth opening, pain in the chewing muscles, and difficulty opening or closing the mouth effectively [3,4]. 

Radiographic evidence of this condition includes erosion of the condyle, growth of bony outgrowths, reduction in joint space, forward positioning of the condylar head, erosion of the head and roof of the glenoid fossa, and flattening of the articular eminence [5].

The goal of the treatment is to obtain a complete remission of the psoriatic arthritis in order to avoid TMJ destruction and ankylosis. We will discuss both symptomatic treatment options and disease modifying antirheumatic drugs (DMARDs).

When it comes to symptomatic treatment for PsA with TMJ involvement, NSAIDs were prescribed in 25 cases out of the 151 cases reported in the literature review of Almășan et al. [6] (treatment was not specified in 97 of the cases), the molecules used were diclofenac sodium 50mg 3 times daily [3], piroxicam 20mg daily [7], and naproxen [8]. Corticosteroids were prescribed in 24 cases, mainly oral prednisolone intake or intra-articular hydrocortisone injections.

DMARDs were prescribed in the cases reported in the same review such as methotrexate (15mg weekly) or leflunomide, and TNF inhibitors such as etanercept 50mg weekly [8], intra articular Infliximab injections [9], and brodalumab 210 mg (subcutaneous injections) [10]. Lamazza et al. reported a case of a 29-year-old male patient with PsA involving the TMJ; he was treated with 25 mg of etanercept subcutaneously twice a week after failing to respond to the conventional DMARDs (ciclosporine) and NSAIDs (naproxen). A follow-up examination after two years revealed complete remission and no damage to the TMJ [8]. Alstergren et al. reported a case of a 62-year-old female patient with PsA involving the TMJ, the traditional DMARDs (methotrexate, chloroquine phosphate, and oral gold) were prescribed for her. The response was positive, but the treatment was stopped due to side effects. Next, etanercept was prescribed; however, it was also stopped because of a possible hypersensitivity reaction. Her symptoms continued despite receiving a systemic infliximab treatment. The symptoms then improved immediately after the first set of bilateral intra-articular infliximab injections (0.5 mL solution of infliximab (Remicade 100 mg) suspended in physiological saline to 10 mg/mL) every sixth week for 36 weeks and a significant improvement persisted throughout the 36 weeks of repeated intra-articular injections [9]. 

Non-pharmacological therapies include physical therapy such as transcutaneous electrical nerve stimulation, occlusal splints, low-level laser therapy, low-level ultrasound therapy, trigger point injections, and arthrocentesis [7].

The mandibular condyle and temporal bone may experience severe bone osteolysis in the advanced stages of the condition, along with condylar resorption and shortening of the mandibular ramus. Complete ankylotic block removal during surgery for TMJ ankylosis is followed by arthroplasty, possibly with an autologous graft between the articular surfaces [6].

## Conclusions

This is a clinical case with two distinctive features: the involvement of the TMJ in PsA and the fact that this involvement can be the first sign of the disease. 

TMJ involvement in PsA is uncommon but can cause a lot of pain and difficulty opening or closing the mouth effectively. It usually goes undiagnosed or untreated. Delay in diagnosis will result in extensive deterioration of the TMJ structures, which will cause persistent discomfort and alter the patient’s quality of life. Hence, the importance of starting the adequate treatment as soon as possible to improve patients' wellbeing. The treatment is based on pharmacological and non-pharmacological measures and involves both the rheumatologist and the maxillofacial surgeon.
